# Alterations of immune response of non-small cell lung cancer with Azacytidine

**DOI:** 10.18632/oncotarget.1542

**Published:** 2013-10-25

**Authors:** John Wrangle, Wei Wang, Alexander Koch, Hariharan Easwaran, Helai P. Mohammad, Frank Vendetti, Wim VanCriekinge, Tim DeMeyer, Zhengzong Du, Princy Parsana, Kristen Rodgers, Ray-Whay Yen, Cynthia A. Zahnow, Janis M. Taube, Julie R. Brahmer, Scott S. Tykodi, Keith Easton, Richard D. Carvajal, Peter A. Jones, Peter W. Laird, Daniel J. Weisenberger, Salina Tsai, Rosalyn A. Juergens, Suzanne L. Topalian, Charles M. Rudin, Malcolm V. Brock, Drew Pardoll, Stephen B. Baylin

**Affiliations:** ^1^ The Johns Hopkins University, School of Medicine, Oncology Center-Hematology/Medical Oncology, Baltimore, Maryland; ^2^ The Johns Hopkins University, School of Medicine, Human Genetics Graduate Program, Baltimore, Maryland; ^3^ Departments of Molecular Biotechnology, Faculty of Bioscience Engineering, Ghent University, Ghent, Belgium; ^4^ The Johns Hopkins University, School of Medicine, Oncology, Baltimore, Maryland; ^5^ GlaxoSmithKline Pharmaceuticals, Cancer Epigenetics and Oncology, Collegeville, Pennsylvania; ^6^ The Johns Hopkins University, Advanced Academic Bioinformatics, Baltimore, Maryland; ^7^ The Johns Hopkins University, School of Medicine, Dermatology and Oral Pathology, Baltimore, Maryland; ^8^ USC Epigenome Center, Keck School of Medicine, University of Southern California, Los Angeles, California; ^9^ The Johns Hopkins University, School of Medicine, Russell H. Morgan Department of Radiology and Radiological Science, Baltimore, Maryland; ^10^ University of Washington and Fred Hutchison Cancer Research Center, Seattle Cancer Care Alliance, Seattle, Washington; ^11^ Memorial Sloan-Kettering Cancer Center, New York, New York; ^12^ The Johns Hopkins University, School of Medicine, Surgery, Baltimore, Maryland

**Keywords:** Non-Small Cell Lung Cancer(NSCLC), Azacytidine, HDAC inhibitor

## Abstract

Innovative therapies are needed for advanced Non-Small Cell Lung Cancer (NSCLC). We have undertaken a genomics based, hypothesis driving, approach to query an emerging potential that epigenetic therapy may sensitize to immune checkpoint therapy targeting PD-L1/PD-1 interaction. NSCLC cell lines were treated with the DNA hypomethylating agent azacytidine (AZA – Vidaza) and genes and pathways altered were mapped by genome-wide expression and DNA methylation analyses. AZA-induced pathways were analyzed in The Cancer Genome Atlas (TCGA) project by mapping the derived gene signatures in hundreds of lung adeno (LUAD) and squamous cell carcinoma (LUSC) samples. AZA up-regulates genes and pathways related to both innate and adaptive immunity and genes related to immune evasion in a several NSCLC lines. DNA hypermethylation and low expression of IRF7, an interferon transcription factor, tracks with this signature particularly in LUSC. In concert with these events, AZA up-regulates PD-L1 transcripts and protein, a key ligand-mediator of immune tolerance. Analysis of TCGA samples demonstrates that a significant proportion of primary NSCLC have low expression of AZA-induced immune genes, including PD-L1. We hypothesize that epigenetic therapy combined with blockade of immune checkpoints – in particular the PD-1/PD-L1 pathway – may augment response of NSCLC by shifting the balance between immune activation and immune inhibition, particularly in a subset of NSCLC with low expression of these pathways. Our studies define a biomarker strategy for response in a recently initiated trial to examine the potential of epigenetic therapy to sensitize patients with NSCLC to PD-1 immune checkpoint blockade.

## INTRODUCTION

Innovative strategies are needed to treat the world's most common cause of cancer death, non-small cell lung cancer (NSCLC) [1, 2]. Less than a quarter of lung adenocarcinomas (LUAD) harbor genetic abnormalities for which targeted therapies have been derived. Early responses are often robust for these but are generally followed by acquired resistance [3, 4]. Lung squamous cell carcinoma (LUSC) has no approved targeted therapies and few effective chemotherapeutic options beyond the first line of therapy. In the current study, we offer a genomically based, hypothesis-driving analysis to suggest a rationale for a novel combinatorial therapeutic approach to efficacious treatments for advanced NSCLC. The backdrop for the present study comes from our initial clinical trials in our Stand up to Cancer project (SU2C) in which patients with advanced, heavily-pretreated NSCLC received a form of “epigenetic therapy” combining low doses of the DNA hypomethylating agent azacytidine (AZA – Vidaza) and the HDAC inhibitor entinostat [5]. Only two of now 65 patients treated to date have had RECIST criteria responses to this therapy alone, but these were very robust and durable (5). A group of patients followed for 8 to 26 months responded to multiple different therapeutic regimens given subsequently, suggesting a “priming” effect of epigenetic therapy (5). Twenty-five percent of these patients with both LUAD and LUSC experienced RECIST criteria responses to their subsequent regimens. These subsequent therapies included not only standard chemotherapies but also immunotherapy targeting the PD-1 immune-checkpoint which when given alone has yielded responses in 16 to 17% of patients with advanced NSCLC [6-8] (Supp. Fig. 1). While the number of patients who have received epigenetic therapy followed by immune checkpoint blockade is small, a clinical trial to evaluate potential sensitization to PD-1 immune checkpoint blockade with epigenetic therapy in patients with NSCLC has now begun.

This trial will be biopsy driven and offer the opportunity to examine hypotheses generated in the present pre-clinical work in order to develop biomarker strategies. In this regard, one of the key therapy agents being employed in the trial is AZA, a nucleotide analog DNA demethylating agent which blocks the activity of all three biologically active DNA methyltransferases (DNMT's) and also triggers degradation of these proteins in the nucleus [9, 10]. With respect to sensitization potential of this drug for immune responses, such targeting of DNMT's is known to induce increased expression of promoter DNA hypermethylated cancer testes antigens and also is reported to up-regulate other individual facets of the tumor immune stimulating profile, including major histocompatibility antigens, and transcription factors *IRF7* and *IRF5* [11-16]. In this regard, we previously reported that elements of such immune pathway activation were produced by low doses of DNA demethylating agents in a genomics based, pre-clinical approach [17]. These studies demonstrated how low doses of AZA, which avoid early, cytotoxic and off-target effects, can provide a memory for a “reprogramming”-like effect on hematopoietic and selected examples of solid tumor cells [17]. We hypothesize in this work that these effects may underlie the fact that significantly lowering doses of DNMT inhibitors in the clinic may account for the markedly decreased toxicity, and significant clinical efficacy, which has led to FDA approval of AZA for myelodysplasia (MDS) [18].

Initially, we have focused our pre-clinical studies for low dose AZA on NSCLC. By first deriving genomic signatures of gene expression responses and DNA methylation for treated NSCLC lines, we observed in most cell lines a complex, multi-faceted up-regulation, involving hundreds of genes of the immune profile of these cells which includes the target of immune checkpoint therapy, the tumor ligand PD-L1. Moreover, using this extensive genomic signature, we have been able to specifically query hundreds of primary NSCLC samples in the Cancer Genome Atlas project (TCGA) for how basal expression of these immune genes and related DNA methylation events group lung cancers. We define a stark clustering of subsets of primary LUAD and LUSC for an “immune evasion” signature, which relates highly to events for low interferon pathway signaling and includes low levels of PD-L1 [20-22]. Low expression of these genes closely matches those up-regulated by AZA treatment of the NSCLC cell lines. We hypothesize that these may be cancers which would benefit from AZA priming together with immune checkpoint therapy and outline a signature that may identify predictive biomarkers from biopsies forthcoming in the current trial.

## RESULTS

### Clinical Data

Six patients who received treatment on a clinical trial of epigenetic therapy for advanced treatment-refractory NSCLC were placed on trials for immunotherapy targeting the PD-1/PD-L1 immune tolerance checkpoint. Of these six patients three have experienced durable partial responses to immunotherapy now ongoing for 14 to 26 months, and the other two had stable disease lasting 8.25 and 8.5 months. (Supp. Fig. 1, Supp. Table 1) For comparison, 41-46% of NSCLC patients on these two trials of immunotherapy alone, one for anti-PD1 and the other for anti-PD-L1 therapy, passed 24 weeks without progression and16-17% had durable partial response rates [6-8].

### AZA Induced Immune Response in Non-Small Cell Lung Cancer Cell Lines

**Figure 1:**
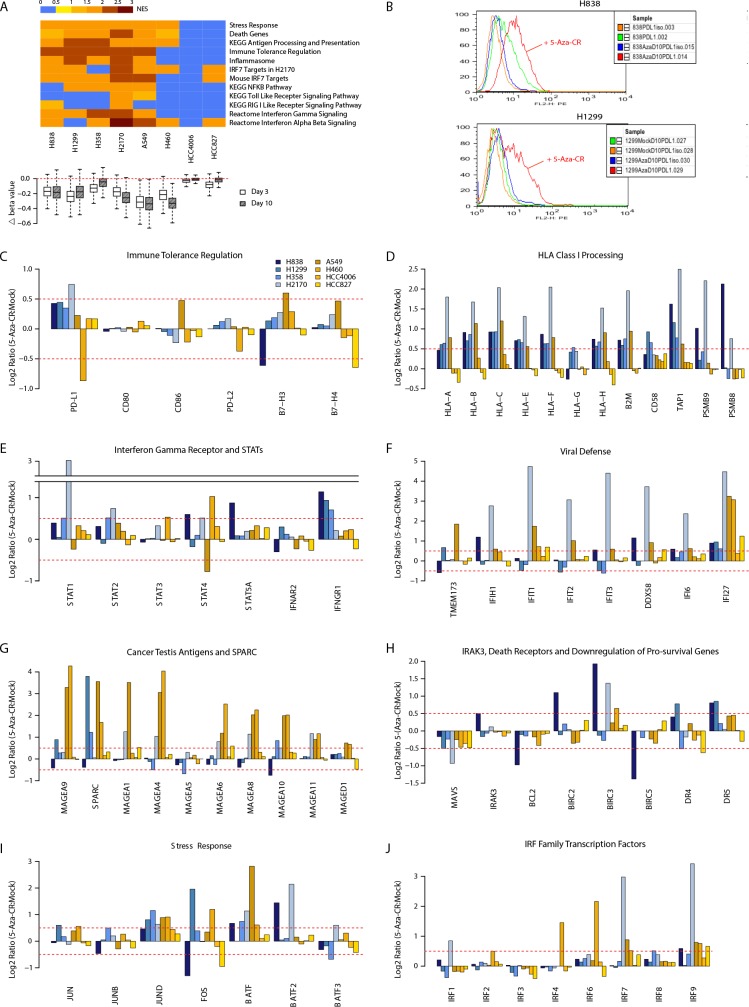
Azacytidine alters gene expression in NSCLC cell lines for multiple immune related pathways (A) Top panel: Gene Set Enrichment Analysis (GSEA) for pathways up-regulated by azacytidine. Normalized enrichment scores are plotted as a heat map. Bottom panel: boxplot showing degree of demethylation in each cell line, as measured by the difference in beta values between the AZA and mock-treated cells immediately after drug withdrawal and 7 days later. (B) FACS analysis shows increased level of cell surface PD-L1 after AZA treatment by day 10 in NSCLC lines H838 and H1299. (C) to (J) AZA-mediated expression changes at day 10 in key genes from pathways outlined in (A). Y axis = Ratio of expression values (log2) of AZA -treated vs. mock-treated cells; X-axis = gene names.

We used our previously validated pre-clinical model to examine how AZA alters expression of key pathways in NSCLC cell lines [17]. Cells were treated in vitro with 500 nM AZA for 72 hours then harvested immediately after withdrawal of drug and again one week later for genome wide methylation and expression studies. To the point of the clinical suggestion that epigenetic therapy may provide sensitization to subsequent immune-checkpoint blockade, we agnostically noted that one or more of the top ten pathways emerging for each cell line were immune related. The genes involved are important to the interaction of both innate and adaptive anti-tumor immunity. As earlier mentioned, other groups have described the ability of AZA to up-regulate individual immune pathway steps relative to assembly of major histocompatibility antigens (HLA Class I), interferon pathway genes, and cancer-testis antigens [11-16]. However, our current analysis reveals a more complex, concordant, broad immune gene signature. Gene Set Enrichment Analysis showed AZA induced up-regulation of multiple immune-related pathways in a manner roughly correlating to the degree of demethylation in response to AZA treatment (Fig. 1A, Supp. Table 2). Each of these components has a demonstrated role in immune tolerance pathways associated with immune checkpoints and immune evasion. Some of these genes have low expression associated with cancer-specific promoter region DNA hyper-methylation, and increased expression after treatment with DNA demethylating drugs [11, 12]. In this regard, it is noteworthy that when compared to normal bronchial epithelial cells, NSCLC is known to exhibit diminished innate immune responses to viral-like stimuli involving intertwined pathways of cell-intrinsic responses to infection and inflammation [11].

### Antigen Presentation

A key step in tumor recognition and killing by cytotoxic T-cells involves recognition of peptides derived from tumor-specific antigens or up-regulated shared antigens bound to HLA Class I antigens expressed by the tumor cells [23]. As recognized by others, AZA increases expression of multiple cancer testes antigens including multiple MAGE family genes, whose expression has been shown to be suppressed by promoter hypermethylation [14, 15] (Fig. 1G). AZA up-regulates not only transcripts of HLA Class I antigens but also a series of genes including, beta-2-microglobulin (*B2M*), *CD58, TAP1*, and the immuno-proteasome subunits *PMSB9* and *PSMB8* which encode proteins required for endoplasmic reticulum processing of, transport to, and anchoring to the cell surface, and recognition of surface HLA class I subunits [24-26] (Fig. 1D). We find generally good correlation between HLA Class I, B2M, CD58, and B7-H3 transcripts and protein on the cell surface by flow cytometry (Supp. Fig. 2). Importantly, mutations potentially contributing to immune evasion have been described in *HLA-A* in a small percentage of LUSC and of *B2M* and *CD58* in other tumor types [26, 27].

### Type I and II Interferon Signaling

A second key issue for immune cell interaction with tumor cells is that, *in vivo*, AZA administration to tumor-bearing mice has been shown to induce antigen processing and presentation genes, particularly when administered with CpG TLR9 agonists, and this is largely attributed to interferon-γ production by lymphocytes [13]. While the lymphocyte-specific γ-interferon is not induced in NSCLC lines with AZA treatment, there is up-regulation of the interferon-γ receptor (*IFNGR1*) as well as of multiple STAT genes, including *STAT1*, the major *IFNGR1* signal transducer (Fig. 1E).

### Programmed Cell Death and Viral Defense

The re-expressed genes in the above mentioned pathways are downstream targets of interferon response pathways in a fashion closely linked to pro-inflammatory and viral defense responses [28-31]. In turn, triggering of these responses can have both tumor repressing activities, such as apoptosis, or tumor promoting events and this paradox has been termed “the dual face” of inflammation [29, 30, 32]. In this regard, we see key subsets of immune related genes that are up-regulated by AZA with potential for inhibiting tumor growth including *IFI27*, which encodes a protein triggering apoptosis in late stages of chronic viral infection[33] (Fig. 1F). Simultaneously, there is down-regulation of the anti-apoptotic gene, *MAVS*, a change which accompanies activation of the RIG I signaling pathway in response to viral challenge [30, 31, 34] (Fig. 1H). Downstream events in viral response include, especially in line H838, simultaneous increases for expression of BIRC family autophagy genes and simultaneous decreases in the anti-apoptotic genes *BCL2* and *BIRC5* (*SURVIVIN*) [35] (Fig. 1H). Indeed, suppression of *SURVIVIN* is known to be triggered by the viral induction of *IRAK3*, which encodes an IL-1 receptor associated kinase [36]. *IRAK3* is, again in H838 cells, up-regulated by AZA concordantly with the death related genes mentioned just above (Fig. 1H). These dynamics are similar to those for colon cancer cells where *IRAK3* is silenced in association with promoter-region DNA hypermethylation and when reactivated by induced demethylation, is associated with *SURVIVIN* down-regulation [36].

### PD-L1 Expression

The key to immune checkpoint therapy is antibody targeting of either the receptor PD-L1 on immune cells and or the ligand PD-L1 on tumor cells [6, 7, 23]. In the clinical trials for immune check point blockade to date involving NSCLC patients, a subset showed no responses when their tumors did not express cell surface PD-L1 [6, 7, 23]. In this regard, when treated with AZA, several NSCLC cell lines up-regulate PD-L1, not only at the transcript level but also at the cell surface protein level (Fig. 1B, 1C). Notably, this AZA increase of *PD-L1* in cell lines is far more consistent than for *PD-L2*, a second dendritic cell/macrophage ligand for the CTL PD-1 receptor, or other checkpoint ligands such as *B7-H3* and *B7-H4* (Fig. 1C). Similarly, *CD80* and *CD86*, the ligands for CTLA4, another therapeutically targeted immune checkpoint receptor, are not altered (Fig. 1C). *PD-L1* expression in tumor cells can either be driven by cell-intrinsic mechanisms or by a process termed adaptive resistance, through interferon-γ signaling and subsequent activation of STAT transcription factors, which we also see induced by AZA (Fig. 1E).

### AZA Alters the Immuno-phenotype of NSCLC Through Its Effect on DNA Methyltransferases

**Figure 2:**
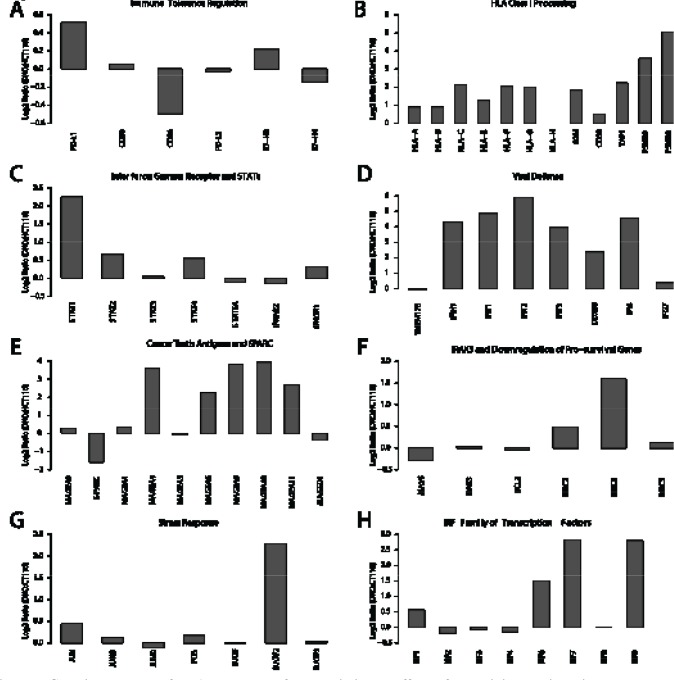
Genetic knock out of DNA Methyltransferases mimics the effects of azacytidine mediated immune pathway up-regulation Gene expression alterations when comparing wild-type HCT116 colon cancer cells to their isogenic DNMT1 and 3B knockout counterpart (DKO). The gene expression differences are given as the log2 ratio of expression in DKO over wild-type HCT116 (Y-axis) and the gene panels, A-H correspond to panels C-J in Fig. 1 for the NSCLC cell lines treated with AZA.

A key issue for all of the above responses is whether these represent attributes of AZA as a targeted therapy. In this regard, this drug, particularly at less toxic doses, specifically targets the three biologically active DNMT's, acting to directly inhibit their catalytic sites and triggering degradation of these proteins in the nucleus [9, 37]. We thus queried how our complex, immune-related, pharmacologic responses compare to simultaneous genetic depletion of two of the three DNMT's. We compared HCT116 colon cancer cells and HCT116 double knock out (DKO) cells that have been genetically disrupted to give severe haplo-insufficiency of DNMT1, and complete absence of DNMT3B, enzymes for DNA methylation maintenance and *de novo* DNA methylation, respectively[38]. These cells have lost the majority of their genome-wide DNA methylation and have de-methylation of many cancer specific, promoter region, DNA hypermethylated CpG islands with corresponding re-expression of genes silenced in the wild type HCT 116 cells [38]. From the standpoint of the present studies, the immune-related expression alterations in DKO versus wild type HCT116 are remarkably similar to the AZA induced changes in NSCLC cells (Fig. 2). We conclude that previously described off target effects of high dose AZA including incorporation into RNA and DNA as an abnormal nucleotide[10] do not appear to be required for the drug's effect that we have defined.

### Up-regulation of Immune Related Transcription Factors by Azacytidine

**Figure 3:**
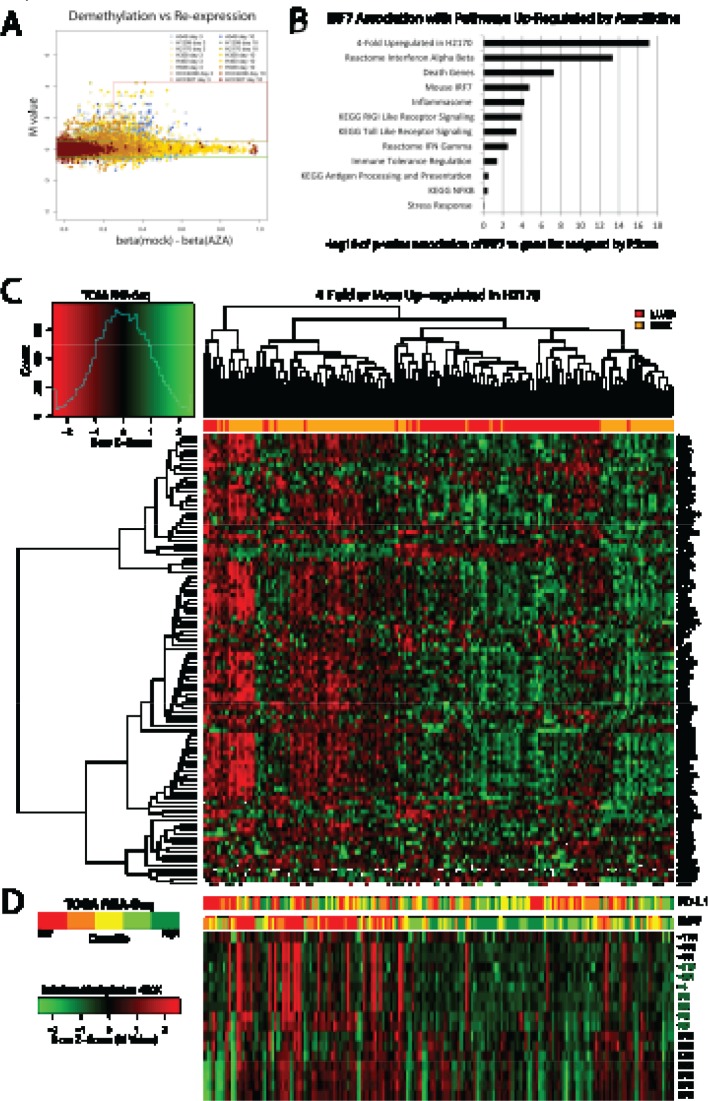
Identification of azacytidine up-regulated transcription factors and interferon signaling related genes, and their clustering of primary Non-Small Cell Lung Cancer in TCGA (A) Identification of genes in Non-Small Cell Lung Cancer cell lines with low basal expression with high basal promoter region DNA methylation which are demethylated and re-expressed with AZA treatment. The red box encompasses genes meeting these criteria which are described specifically in methods. Among these, *IRF7*, a key immune-related transcription factor, was up-regulated in multiple cell lines. (B) Pathways up-regulated in NSCLC cell lines in response to AZA are enriched for *IRF7* targets as determined by PScan analysis (-log_10_ of p-values) and gene set enrichment analysis. (C) Heat map of RNA-Seq expression levels in primary lung cancers from TCGA database for genes 4-fold or more induced by AZA in the LUSC cell line H2170, the cell line with the greatest degree of IRF7 up-regulation. Top bar: red indicates LUAD and orange indicates LUSC samples. Genes used in the heat map are listed in supplemental table 4. (D) Bar panels show expression of *PD-L1* and *IRF7* in five quantile intervals (red for lower and green for higher expression). Heat map immediately below *IRF7* expression bar shows corresponding Infinium platform DNA-methylation levels (Z-scores, red for more and green for less methylated) across the promoter region. Positions relative to transcription start site are shown to the right. CpG-island probes are labeled in green. Sample order in bar plots and methylation heat map is maintained from the main heat map.

In order to find specific genes re-expressed in response to AZA which may be driving immune-related changes we extensively filtered our genome wide expression and methylation data from cell line experiments to identify transcription factors meeting criteria of epigenetically re-expressed genes. Approximately 300 genes with high baseline promoter region CpG island methylation, promoter demethylation of 25% or more after treatment, and expression increased by log_2_ 0.5 (1.4-fold) or greater after treatment (Fig. 3A, Supp. Table 3). Nearly 17% are in an interferome database[39] (http://www.interferome.org), and 19% are transcription factors [39, 40]. The transcription factor *IRF7* has been reported by others to be hypermethylated in cancer, as it is in our NSCLC line with the lowest basal expression [11, 40-42]. It is up-regulated in response to AZA in several cell lines, most prominently in the LUSC cell line H2170, showing a 9-fold increase (Fig. 1J). IRF7 is an upstream activator of functions in cellular pathways recognizing the virus response element VRE-A to increase transcription of genes involved in type 1 IFN signaling [11]. There is a significant association of *IRF7* transcription targets with genes driving several of our GSEA enrichment scores for the immune pathway alterations observed in response to AZA (Fig. 3B).

### Immune-Phenotypes within Histologies in The Cancer Genome Atlas

**Figure 4:**
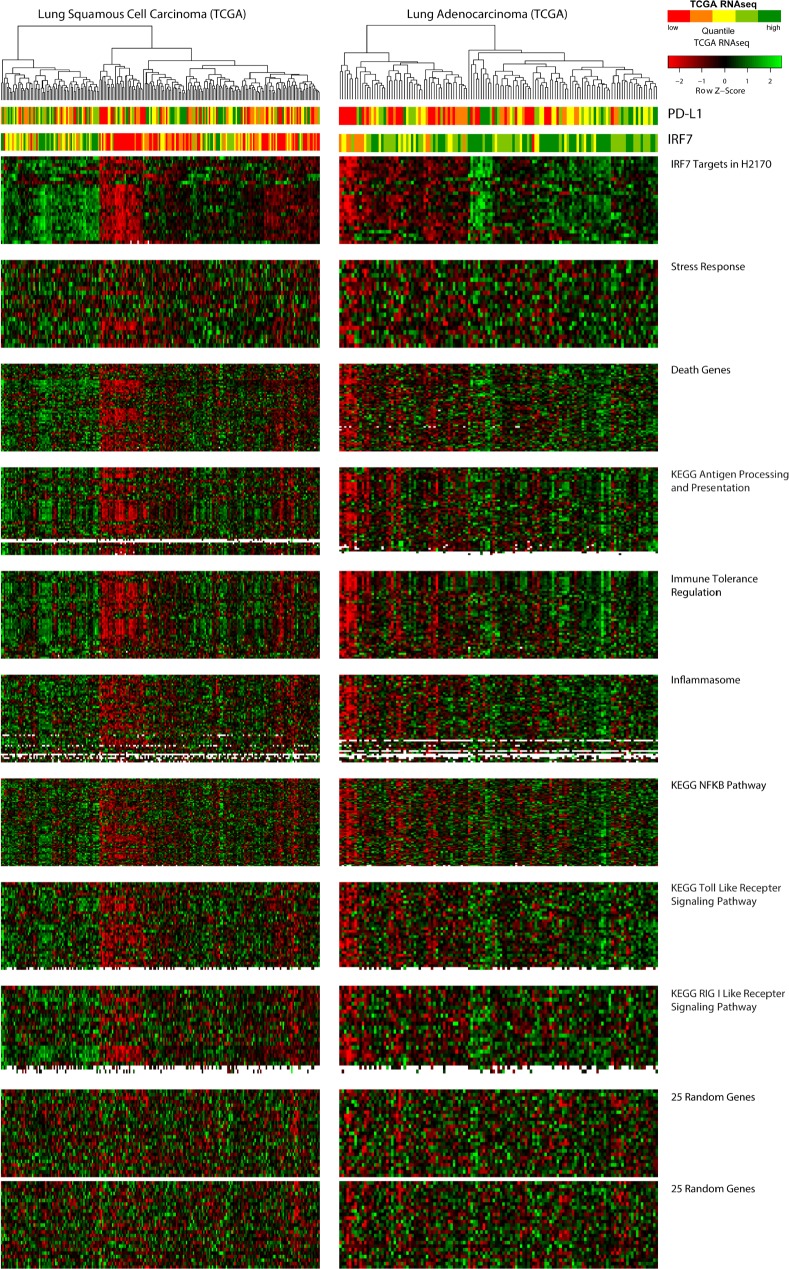
Relationship of azacytidine-induced, immune-related pathways to primary lung tumors grouped by expression of IRF7-associated genes TCGA samples are ordered by unsupervised clustering based on genes highly up-regulated in H2170, which are enriched for IRF7-targets, represented in the topmost heat map. Order of samples is maintained in all lower heat maps. PD-L1 and IRF7 expression are depicted in the top bar panels as in figure 3D. Supplemental Table 5 table shows the overlaps of genes from each pathway represented in the heat maps. That the observed clustering pattern is not due to chance or batch effect is demonstrated using random sets of 25 genes shown in the bottom two panels.

From our analysis suggesting *IRF7* to be a potentially important cancer-specific hypermethylation induced down-regulation event, we sought to create a list of functionally derived genes closely associated with its re-expression. Examining H2170, the LUSC cell line with the greatest up-regulation of *IRF7* we hypothesized that other genes highly up-regulated in this cell line might be targets of this transcription factor (Fig. 1J). Filtering expression array data, 114 genes where found to be 4-fold or more up-regulated in response to AZA in the H2170 (Supp. Table 4). The association of this functionally derived gene list with *IRF7* is confirmed by PScan analysis (p = 7.6 e −18) (Fig. 3B). These data suggest that IRF7 silencing by DNA methylation in tumors could result in suppression of immune-regulatory genes important for the surveillance of tumors by cytotoxic immune mechanisms. Other studies have reported an immune-evasion signature dependent on IRF7 in breast and melanoma [40, 43]. To test if such relation between IRF7 and immune-regulatory genes exist in primary LUAD and LUSC tumors, we analyzed the expression of these genes as a function of IRF7 expression, and its promoter methylation status. We found that low expression of these genes describes a subgroup, particularly among LUSC, in TCGA samples which clusters tightly with high promoter region DNA methylation and low expression of *IRF7* (Figs. 3C, 3D, and 4). Finally, expression levels of *PD-L1*, the key tumor ligand targeted in the anti-checkpoint immunotherapy trials, tracks quite well with the above immune evasion signature in subgroups of not only LUSC, but also LUAD, as especially well visualized in heat maps for individual immune related pathways, which each track closely with an immune evasion signature in the LUSC and LUAD, TCGA samples (Fig. 4).

## DISCUSSION

In the present work, we have used an in-vitro model to derive a pre-clinical understanding of the immunomodulatory effects of clinically relevant doses of AZA in NSCLC that may underpin its potential to “prime” for subsequent response to PD-1 pathway blockade. We characterize an AZA induced expression signature of immune genes and pathways in NSCLC known to play a role in the down-regulation of immune surveillance of cancer. However, concomitant with induction of the immune genes comprising both innate and adaptive immunity is the up-regulation of a primary immune inhibitory ligand, PD-L1. Our data therefore suggest a mechanism by which epigenetic therapy might improve the outcome of treatment of patients with NSCLC with PD-1/PD-L1immune checkpoint blockade. By matching these basal gene expression and DNA methylation patterns, including that of a core interferon pathway transcription factor, IRF7 in the TCGA project, we extrapolate our in vitro AZA-induced gene signature to hundreds of primary NSCLC cancers. These results suggest that a major effect of AZA treatment is the alteration of tumor immune inducing, pathways that could lead to susceptibility of tumor cells themselves to immune attack by T cells. In particular, because the inhibitory ligand PD-L1 is up-regulated by AZA in our cell lines, and subsets of primary tumors have concordant low-expression of AZA induced immune genes and PD-L1, we suggest that combination of epigenetic therapy and PD-1 pathway blockade might produce a synergistic anti-tumor response.

Our findings provide a basis for biomarker approaches that we will test in a just initiated trial for patients with advanced LUAD and LUSC, aimed at validating the promise for sensitization by epigenetic therapy to immune checkpoint therapy. If we continue to see robust patient efficacy, our data may prove key to determining which individuals are likely to benefit from the epigenetic therapy approaches we are testing in clinical trials by evaluating gene panels for expression and DNA methylation in pre-and post- drug administration biopsies.

## MATERIALS AND METHODS

### Clinical Data

Institutional review board approved informed consent signed by each patient allowed the collection of clinical data following treatment on trial with epigenetic therapy. Relevant data were obtained by chart review. Representative images demonstrating responses to therapy were obtained from computed tomography series employed in the assessment of patient responses to anti-PD1 or anti-PD-L1 directed immune-checkpoint therapy. Assessment of response to treatment was performed by a single reference radiologist who employed (RECIST 1.0) to generate measurements for target lesions to be followed over the course of therapy. Change in target lesions from baseline (%) is calculated by summing the diameter of all target lesions at each radiographic tumor evaluation and calculating percentage change at a given time point ([(Target Lesion SumTimepoint X/Target Lesion SumBaseline)−1]*100).

### TCGA Samples

Level 3 RNA-Seq data (Illumina HiSeq RNA-Seq platform, Illumina, Inc., San Diego, CA, USA) were downloaded for 353 NSCLC samples (129 LUAD / 224 LUSC) and 54 adjacent non-tumor lung tissue samples from the TCGA Data Portal (https://tcga-data.nci.nih.gov/tcga/). Similarly, level 1 DNA methylation data (Illumina Infinium HumanMethylation450 BeadChip, Illumina, Inc., San Diego, CA, USA) were downloaded for 353 NSCLC samples (222 LUAD / 149 LUSC) and 74 adjacent non-tumor lung tissue samples. Among these, data for 174 NSCLC samples (80 LUAD / 94 LUSC) and 21 adjacent non-tumor lung tissue samples were available on both of the above platforms.

### RNA-Seq Data Analysis

We used TCGA level 3 RNA-Seq data already normalized and quantified at gene levels, and presented as RPKM values (Reads Per Kilobase per Million mapped reads). To construct heat maps: 1) Values of 0 (indicating no reads observed for a gene) in the RPKM data were set to NA; 2) the remaining RPKM values were log 2 transformed; 3) genes from X and Y chromosomes were removed; and 4) heat maps were made using the “heatmap.2” function in “gplots” package from CRAN[44]being centered and scaled in the row direction, and using the default functions for computing distance and hierarchical clustering (or being specifically ordered in column according to the order of other heat maps). Expression spectrums for individual genes were displayed in five quartile intervals following the order of associated heat maps of the RNA-Seq data.

### Infinium DNA Methylation Data Analysis

TCGA level 1 DNA methylation data contain raw binary intensity data files. Raw data files were imported into R (http://www.r-project.org) to calculate beta values (beta value Infinium = M / [U + M], M: mean intensities of the Methylated bead type, U: mean intensities of the Unmethylated bead types), M values (M value Infinium = log 2 [M / U]) and detection p-values (calculated by comparing probes to negative control probes to determine if signals are significantly different from the background) using the “methylumi” package from Bioconductor [45]. Beta values and M values for probes with detection p-value > 0.05 were considered not significantly different from background and were masked as NA. TCGA methylation data were first assessed for batch effects by principle component analysis (PCA) on the M values. To accomplish this, data points from X chromosome and Y chromosome as well as data points that are associated with SNPs (Single Nucleotide Polymorphisms) were removed, and the first two principle components are used for plotting.

Spearman's correlation coefficients between methylation (beta value of probe, Illumina Infinium HumanMethylation450 BeadChip) and gene expression (RPKM value of gene, Illumina HiSeq RNA-Seq platform) were calculated using TCGA samples with available data on both platforms. For a particular gene, only methylation probes that have a negative Spearman's correlation coefficient and a adjusted p-value (FDR) for the coefficient < 0.01 were considered informative and their relative distances to the corresponding transcriptional start site (TSS) of the genes were calculated from genomic coordinates obtained from the UCSC genome browser (http://genome.ucsc.edu). Heat maps of the M values of informative probes were made using the “heatmap.2” function in “gplots” package from CRAN[44] being centered and scaled in the row direction, and ordered according to the associated heat maps of the RNA-Seq data in column and to the relative distances to TSS in row.

For in vitro DNA methylation values, DNA was extracted from cell lines that were either untreated or treated with AZA at day 3, at the end of treatment, and day 10 (7 days post end of treatment) and analyzed by the Illumina Infinium HumanMethylation450 BeadChips (Illumina, Inc., San Diego, CA, USA). Raw data were imported into R using the “methylumi” package from Bioconductor [45]. Data points for probes with detection p-value > 0.05 were masked as NA. Δ beta values (Δ beta value = beta value AZA – beta value Mock) were calculated and used to make boxplots. Heat maps were made similarly like those for the TCGA data using informative probes defined by the TCGA data.

### Expression Microarray Data

For in-vitro RNA extracted from cell lines treated with AZA, analyses were done at exactly the same time points as for DNA methylation above. Analyses from wild type colon cancer, HCT116 cells, and genetic knockout counterparts for DNA methyltransferases (DKO cells) were also performed. Expression microarrays were carried out using Agilent Human 4× 44K expression arrays (Agilent Technologies, Santa Clara, CA, USA, Cat#: G4112F). Within-array and between – array normalization was performed using Loess and Aquantile normalization, respectively[46]. Median of the M values (M value Expression = log 2 [AZA / Mock] OR log 2 [DKO / HCT116]) was determined for multiple probes associated with the same gene.

### Gene Set Enrichment Analysis (GSEA)

For each of the eight lung cancer cell lines (H838, H1299, H358, H1270, A549, H460, HCC4006, HCC827) a ranked gene list was created (genes were sorted by decreasing M value). These eight ranked gene lists were entered in the GSEA tool[47, 48]and the enrichment of both Kegg [49] and Reactome[50] pathways in these lists was calculated (default parameters). A gene set was selected when it was enriched in any of the eight cell lines (p value < 0.05 and false discovery rate < 0.25). The normalized enrichment scores (NES) for the gene sets in each cell line were used to create the heat maps. When a certain gene set was not significant in a cell line, it was assigned a NES of 0.

### Transcription Factor Analysis

Expression and methylation data were analyzed to find genes whose re-expression was linked to demethylation after AZA treatment. Genes were selected based on a set of cut-offs, both for the methylation and expression values: A gene was considered to be re-expressed when at day 3 or day 10 the median M value of all the probes linked to that gene was higher than 0.5. Infinium probes were analyzed separately at their distances from the transcription start site for each gene examined. For a probe to be called demethylated, it had to have a beta value higher than 0.5 in the mock treatment and a difference in beta value between mock and AZA treatment had to be at least 0.25. Only probes that were associated with a CpG island and that were located within 1000 bp upstream and 1000 bp downstream of the transcription start site were used in the analyses. The probes that passed these filters were validated using the TCGA methylation and expression data (see the definition of informative probes in the “Infinium DNA Methylation Data” section of Methods). Only genes that had an expression-methylation correlation value < −0.25 and a false discovery rate < 0.05 were retained. To better understand the biological implications of the re-expressed genes, the gene lists were searched for transcription factors. Two human transcription factor lists obtained from Ravasi et al [51]and Vaquerizas et al [52, 53] were combined and the resulting list was matched to the lists of demethylated and re-expressed genes. The targets of IRF7 from the list of genes that are 4-fold or more up-regulated in H2170 by AZA were similarly identified using the TranscriptomeBrowser database [54].

### Flow Cytometry Methods (FACS)

Frozen cells were thawed in 37 degrees Celsius and washed once with flow-washing buffer. Aliquots of single-cell suspension were then stained with fluorescent-labeled antibodies for 15 minutes at room temperature. Each sample was washed twice and re-suspended in flow-washing buffer and analyzed by FACSCalibur. The following antibodies were used: CD274 (12-5983-42 Ebiosciences), HLA abc (12-9983-42 Ebiosciences), CD276(331606 Biolegend), CD119(558934 BD), B2 microblogumin(551337BD), CD58(555921BD). Changes between AZA treated and mock cells are calculated using mean fluorescence intensities (MFI) and the formula log2([(MFI_antibody, treated_)−(MFI_isotype, treated_)]/ [(MFI_antibody, mock_)−(MFI_isotype, mock_)]).

### PSCAN

PSCAN (http://159.149.160.51/pscan/) [54] is an online software tool that predicts the association of user defined gene-lists with transcription factors by scanning promoter sequences of co-regulated or co-expressed genes looking for over- or under-represented motifs. RefSeq IDs of the gene lists were obtained from BioMart (http://www.biomart.org/) and analyzed in PSCAN. Scanned promoter region was −450 to +50 base pairs around the transcription start site and employing TRANSFAC as the database for co-regulated or co-expressed genes.

## Supplementary Figures and Tables




