# Supplementary material for: Alterations of immune response of non-small cell lung cancer with Azacytidine
Source: Oncotarget. 2013 Oct 25;4(11):2067–79. doi: 10.18632/oncotarget.1542 (PMC3875770; doi:10.18632/oncotarget.1542)
Supplement: Supplementary file 1 [file oncotarget-04-2067-s001.pdf]

SUPPLEMENTAL FIGURES

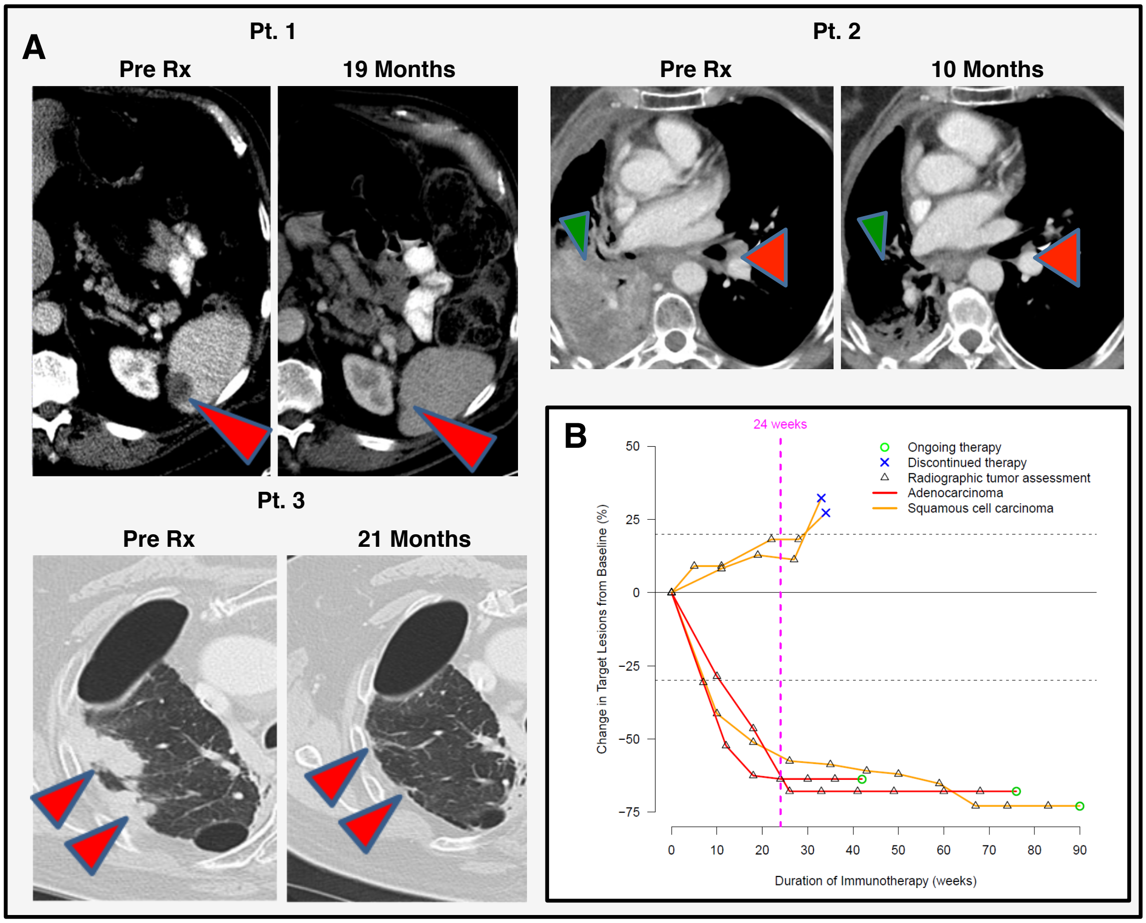

Supplemental Figure 1: Outcomes for five patients treated with immune checkpoint immunotherapy after epigenetic therapy. (A) Scans for 3 patients (Pt.) with RECIST criteria responses to either PD-1 or PD-L1 therapy. All scan interpretations were performed by a single radiologist and lesions used to measure tumor shrinkage between pre- and during immunotherapy at specified times are shown by red arrows (metastasis in the spleen- Pt.1; lung tumor lesions- Pt.2; lymph node in right central chest with metastases –Pt. 2. Green arrow denotes large area of the right lung collapsed behind airway obstruction by tumor and resolving by the 10 month period after immunotherapy. (B) Spider plot of sequential scan measurements (Y-axis) of lesions relative to time of treatment initiation with anti-PD-1 or anti-PD-L1 shown in panel (A) by weeks (X-axis) with a decrease of 30% qualifying as RECIST criteria response (green circles). Blue crosses indicate tumor increase of > 20% qualifying as disease progression. The 24 weeks point denoted by the dashed vertical line represents a duration of treatment after which disease stabilization is conventionally considered to represent clinical benefit.

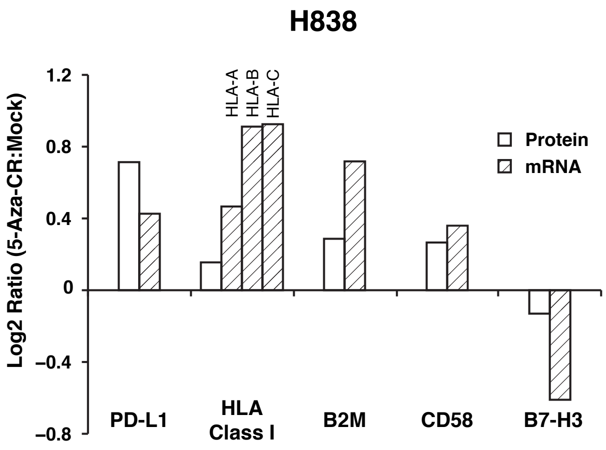

Supplemental Figure 2: Comparison of expression array data to flow cytometry for select cell surface proteins in H838. Clear bars represent the log2 ratio of mean fluorescence intensity of AZA over mock treated cells. Hashed bars represent the M-values of expression array (log2[AZA:Mock]). For HLA Class I, the antibody used in flow cytometry does not discriminate subtypes of class I molecules. Individual class I molecule subtype transcript data are available from the Agilent array platform and is presented. Changes between AZA treated and mock cells are calculated using mean fluorescence intensities (MFI) and the formula $\log2([(MFI_{antibody, treated})-(MFI_{isotype, treated})]/ (MFI_{antibody, mock})-(MFI_{isotype, mock})]$
