# Supplementary material for: Alterations of immune response of non-small cell lung cancer with Azacytidine
Source: Oncotarget. 2013 Oct 25;4(11):2067–79. doi: 10.18632/oncotarget.1542 (PMC3875770; doi:10.18632/oncotarget.1542)
Supplement: Supplementary file 2 [file oncotarget-04-2067-s002.docx]

Alterations of immune response of non-small cell lung cancer with Azacytidine - Wrangle et al

**Supplementary Tables**

[**Supp Table 1-2.xlsx**](Supp%20Table%201-2.xlsx)

[**Supp Table 3-5.xlsx**](Supp%20Table%203-5.xlsx)
